# Genetics and Evolution of Abiotic Stress Tolerance in Plants

**DOI:** 10.3390/genes13081380

**Published:** 2022-08-01

**Authors:** Patrizia Galeffi

**Affiliations:** Italian National Agency for New Technologies, Energy and Sustainable Economic Development, ENEA, Casaccia Research Center, 00123 Rome, Italy; patrizia.galeffi@enea.it

Now more than ever, the understanding of the genetics and evolution of the gene mechanisms and the networks of different molecular pathways acting on plant abiotic stress tolerance has an important role in the finding of new solutions and approaches mitigating the effects of global climate changes, thus contributing to a correct equilibrium among human needs, food security and human health and wellbeing.

Science and research have a major role in this context.

Scientists have the important task of increasing the level of knowledge, which is still not sufficient, in the complex area of plant genetics and genomics, particularly in relation to genes responsive to specific abiotic stresses (such as drought, salts or heat) and their inducible promoters, and to various gene expression control and modulation mechanisms, including alternative splicing, micro-RNA interference, post-transcriptional mRNA decay, and post-translational protein degradation. At the same time, evolution has played a key role in the establishment of the current plant molecular traits, so that a better understanding of the genetic diversity producing different alleles, adaptation, phylogenesis, and evolution of genomes and gene families can be translated and applied as a tool for developing new tolerant plant varieties able to satisfy our needs, in terms of food security, protection of the planet, and conservation and recovery of natural resources, such as water and soils.

I found that reading the various articles published in the current Special Issue represented an exciting virtual tour through plant molecular responses to various environmental stresses. To put together this book, I have divided the 10 papers (9 original research manuscripts and 1 review) into three groups and identified a common thread linking one article to another to accompany the reader on this virtual tour through plant genetics and molecular responses to abiotic stresses. 

The first group of papers consists of two interesting studies based on field trials: the first one written by Marco Dettori and colleagues and focused on the development of a method to predict the ability of different varieties of durum wheat to respond to drought/heat stress. The prediction is based on a statistical approach and mathematical model using a large number of field data collected over 10 years of field experiments. The second paper, written by Arun K. Joshi and his team, is based on a large quantity of field trial data collected over two seasons, using more than 3000 varieties of wheat grown in India and other Asiatic regions, where the climate produces a strong negative effect on the grain yield and other yield-related traits. Their agronomic evaluations relate to the molecular effects of the *Vrn-1* gene, the *Ppd-1* gene and their alleles on complex traits, such as flowering time and photoperiod sensitivity. Their research suggests how to avoid the damage due to heat/drought stress by using the large numbers of allele combinations involved to regulate growth habits and achieve optimal adaptation.

The second group includes two other papers: one written by Arianna Latini et al. regarding functional genomics research into the expression profiles of a drought-responsive transcription factor gene *(DRF1)* of durum wheat in fields under full- and reduced-irrigation conditions. This article reports the expression profiles of the three *TdDRF1* gene transcripts, from durum wheat genotypes during different plant growth stages. In addition, the expression profile of one putative target gene (*Wdnh13*) is investigated and some analogies are found. The results highlight significant differences in the molecular patterns and suggest that the expression profile of this stress-related transcription factor gene is genotype dependent. Furthermore, a statistical association between the expression of *TdDRF1* transcripts and agronomic traits is also revealed, with significant differences among genotypes.

The other article, written by Giuseppe E. Condorelli et al., focuses on a GWAS work which reveals 15 QTLs (Quantitative Trait Loci) for Osmotic Adjustment (OA) found to have a high capacity when positively associated with Relative Water Content and/or Chlorophyll Content, and a low capacity when negatively associated with leaf rolling, thus indicating the beneficial effect of the OA-QTLs on the global plant water status.

These two articles, moreover, succeed in the difficult task of bringing together genomics studies with extensive field experiments.

The third group of papers consists of five articles that span from transcriptomics to metabolomics and cover the molecular behavior of different genes taken as examples. The article, authored by Deheng Yao et al., reports a comparative transcriptome analysis of rosemary suspension cells carried out at different concentrations of Methyl Jasmonates (MeJA). A large number of differentially expressed genes were identified, with totals of, respectively, 7836, 6797, and 8310 genes from the lowest to the highest concentration. This study demonstrates a strong involvement of these genes in the regulation of the biosynthesis of active compounds relevant to the physiological response to stresses. 

The article written by Ashraful Islam et al. analyzes the Trehalose-6-Phosphate Phosphatase (*TPP*) genes in *Triticum aestivum* L. and identifies their gene family, composed of 31 genes organized in 5 clades, also found in *Hordeum vulgare, Brachypodium distachyon,* and *Oryza sativa,* and provides evidence of an evolutionary status. These TPP genes are involved in trehalose-metabolism, which has a role in stress tolerance. Most of the results achieved in this study were obtained using bioinformatics and silico tools, which helped in the prediction and assessment of gene structure and functioning.

The article written by Joran K. Waititu et al. describes transcriptome profiling in *Zea mays* and reveals different key cold-responsive genes, transcription factors and metabolic pathways regulating cold stress tolerance, using a comparative approach on 24 cold-tolerant and 22 cold-sensitive inbred lines affected by cold stress at the seedling stage. Valuable insights arise from the identification of 2237 differentially expressed genes (DEGs), one-third of them being annotated.

The study written by Basmah M. Alharbi et al. focuses on the biochemical and molecular effects induced by Triacontanol (TRIA) in acquired drought stress tolerance in rice. After 10 days of plant exposure to drought stress, data regarding photosynthetic pigments, stomatal conductance and photochemical efficiency were collected and analyzed. The cultivar Giza 177 (*Oryza sativa* L.) was found to be sensitive to drought stress. TRIA treatment enhanced both growth and acquired plant tolerance, by increasing the content of free amino acids and sugars, improving the ability of plant tissues to retain more water under scarcity conditions and regulating Aquaporins (AQPs), which are a class of intrinsic proteins playing an important role in transmembrane water transport.

The article written by Pierre Jacob et al. focuses on the *Heat Shock Factor A2* (*HSFA2*) regulator and co-regulated genes involved in multiple environmental stress responses required for stress acclimation. They identified 43 genes strongly co-regulated with *HSFA2* during multiple stresses. A motif of the site II element (SIIE) found in the promoters of these genes was identified to be closely related to R2R3-MYB transcription factors TT2 and MYB5. The over-expression of these factors was also investigated by transient transformation to evaluate their involvement in heat stress tolerance. 

Last but not least, the review written by Rahat Sharif et al. addresses the problem of the HD-ZIP gene family by trying to clarify, through a comprehensive literature analysis, the potential roles of this gene family in improving plant growth and regulating stress-responsive mechanisms in plants. There are many interesting insights and the reader will be positively impressed. 

This Special Issue was “not easy to manage”, as it encompasses many aspects of different genes with the role of regulating and modulating the expression profiles of themselves and other downstream genes in very complex genetic, molecular, and metabolic networks. 

The common thread linking all these articles together is the challenging study of the big data coming from genomics, transcriptomics, and metabolomics with the aim of highlighting the various, still unknown, mechanisms and regulatory systems, that are underlying the abiotic stress tolerance responses in plants.

I wish to thank very much all the authors and their co-authors for their valuable contributions, their sharing of their scientific work, their experience, and their commitment to this Special Issue, which was a “difficult task” but, in the end, proved a success in terms of acquired knowledge, new ideas, and future perspectives.

Furthermore, I wish to thank all the editorial staff and the MDPI group for their tireless assistance to me and all the authors, resulting in the publication of our Special Issue in a captivating and interesting book with a certain original appeal.

Finally, good reading to all!

